# Supplementary material for: Adaptive enrichment trial designs using joint modelling of longitudinal and time-to-event data
Source: Stat Methods Med Res. 2024 Oct 16;33(11-12):2098–114. doi: 10.1177/09622802241287711 (PMC11577695; doi:10.1177/09622802241287711)
Supplement: sj-pdf-1-smm-10.1177_09622802241287711 - Supplemental material for Adaptive enrichment trial designs using joint modelling of longitudinal and time-to-event data [file sj-pdf-1-smm-10.1177_09622802241287711.pdf]

Supplementary materials for Adaptive enrichment trial designs using joint modeling of longitudinal and time-to-event data

Abigail J. Burdon, Richard D.Baird and Thomas Jaki

July 4, 2024

A Alternative joint model and estimation methods

A.1 Joint model with treatment effect on the biomarker

As described in the main text, it may be desirable to have a joint model which accommodates a treatment effect in the biomarker trajectory. This model and analysis method are now described.

Let the times of the measurements of the longitudinal data for patient i in subgroup $j = 1, 2$ be denoted by $v_{ji1}, \dots v_{jim_{ji}}$, then $X_{ji}(v_{jis})$ is the true value of the biomarker at time v_{jis} and $D_{ji}(v_{jis})$ is the observed value of the biomarker. Suppose that ψ_{ji} is the indicator function that patient i in subgroup $j = 1, 2$ receives the experimental treatment. Let θ_j and γ_j be scalar coefficients. The longitudinal data model takes the form

$$\begin{aligned} X_{ji}(v_{jis}) &= b_{0ji} + b_{1ji}v_{jis} + b_2 v_{jis}\psi_{ji} \\ D_{ji}(v_{jis}) &= X_{ji}(v_{jis}) + \epsilon_{ji}(v_{jis}) \\ \begin{bmatrix} b_{0ji} \\ b_{1ji} \end{bmatrix} &\sim N\left(\begin{bmatrix} \mu_{1j} \\ \mu_{2j} \end{bmatrix}, \begin{bmatrix} \phi_{1j} & \phi_{12j} \\ \phi_{12j} & \phi_{2j} \end{bmatrix} \right) \end{aligned} \quad (1)$$

where $\mathbf{b}_{ji} = (b_{0ji}, b_{1ji})$ is a vector of patient specific random effects and $\epsilon_{ji}(v_{jis})$ is the measurement error. We make the assumptions that $\epsilon_{ji}(v_{jis})|\mathbf{b}_{ji} \sim N(0, \sigma_j^2)$ for $s = 1, \dots, m_{ji}$ and $\epsilon_{ji}(v)$ and $\epsilon_{ji}(v')$ are independent for $v \neq v'$.

For the survival model, let $h_{ji}(t)$ be the hazard function for patient i in subgroup j and h_{oj} be the baseline hazard function for subgroup j. Then the survival submodel takes the form

$$h_{ji}(t) = h_{0j}(t)\exp\{\gamma_j X_{ji}(t) + \theta_j\psi_{ji}\} \qquad \text{for } j = 1, 2. \quad (2)$$

A.2 5-year restricted mean survival time (RMST)

For the joint model of Equations (1)—(2), the coefficients b_{2j} and θ_j are two treatment parameters where θ_j is the direct effect of treatment acting on survival and b_{2j} is the indirect effect. We require a single one dimensional test statistic that summarises the overall effect of treatment and we propose using the restricted mean survival time (RMST) to do so.

Royston and Parmar[9] define RMST as the area under the survival curve up to time t^*. The value of t^* is fixed at the design stage and we shall discuss our choice. Let $\boldsymbol{\eta}_j$ be the $p \times 1$ vector of all parameters in the joint model in subgroup j. Suppose that F_{0j} and F_{1j} are time-to-failure random variables for patients on the control and experimental treatment arms respectively and that $S_{0j}(t; \boldsymbol{\eta}_j)$ and $S_{1j}(t; \boldsymbol{\eta}_j)$ are the corresponding survival functions integrated over any patient specific random effects. Then the difference in RMST between treatment groups is

$$\Delta_j(t^*; \boldsymbol{\eta}_j) = \mathbb{E}[\min(F_{1j}, t^*)] - \mathbb{E}[\min(F_{0j}, t^*)] = \int_0^{t^*} \left[S_{1j}(t; \boldsymbol{\eta}_j) - S_{0j}(t; \boldsymbol{\eta}_j)\right] dt. \quad (3)$$

Most commonly, non-parametric methods are employed for estimating $\Delta_j(t^*; \boldsymbol{\eta}_j)$ when the proportional hazards assumption does not hold since the estimator is robust to model misspecification. Our motivation for using RMST is to

find a test statistic summarising the effect of two treatment parameters. Hence we shall focus on the parametric RMST estimate which is now described.

Let the cumulative hazard function for patient i in subgroup j be $H_{ji}(t;\boldsymbol{\eta}_j)$. The control and experimental treatment survival functions integrated over the random effects are

$$S_{0j}(t;\boldsymbol{\eta}_j) = \int_{-\infty}^{\infty} \exp\{-H_{ji}(t;\boldsymbol{\eta}_j, \psi_{ji} = 0\} f(b_{0ji}, b_{1ji}) db_{0ji} db_{1ji}$$
$$S_{1j}(t;\boldsymbol{\eta}_j) = \int_{-\infty}^{\infty} \exp\{-H_{ji}(t;\boldsymbol{\eta}_j, \psi_{ji} = 1\} f(b_{0ji}, b_{1ji}) db_{0ji} db_{1ji}$$

where $f(b_{0ji}, b_{1ji})$ is the probability density function for the normal distribution of the random effects. Gauss-Hermite integration can be used to efficiently calculate the integrals over b_{0ji} and b_{1ji}. The survival functions are then substituted into Equation (3) to calculate $\Delta_j(t^*;\boldsymbol{\eta}_j)$. Rizopoulos [8] presents the full likelihood function for the joint model from which we can obtain the MLE $\hat{\boldsymbol{\eta}}_j^{(k)}$ at analysis k. An estimate for the treatment difference is given by $\Delta_j(t^*;\hat{\boldsymbol{\eta}}_j^{(k)})$ for $k = 1, \ldots, K$.

The delta method by Doob [1] is used calculate the variance of the parametric RMST estimate. We have that $\hat{\boldsymbol{\eta}}_j^{(k)}$ has the same dimensionality as $\boldsymbol{\eta}_j$, a $p \times 1$ vector. Let $\Sigma_j^{(k)}$ be the $p \times p$ covariance matrix of the MLE $\hat{\boldsymbol{\eta}}_j^{(k)}$ at analysis k, then we have that $n^{1/2}(\hat{\boldsymbol{\eta}}_j^{(k)} - \boldsymbol{\eta}_j) \xrightarrow{d} N(\mathbf{0}, \Sigma_j^{(k)})$. The information level at analysis k for the difference in RMST between treatment arms is given by

$$\mathcal{I}_j^{(k)} = n_j \left(\left[\partial \Delta_j(t^*;\hat{\boldsymbol{\eta}}_j^{(k)})/\partial \boldsymbol{\eta}_j\right]^T \Sigma_j^{(k)} \left[\partial \Delta_j(t^*;\hat{\boldsymbol{\eta}}_j^{(k)})/\partial \boldsymbol{\eta}_j\right] \right)^{-1}$$

where $\partial \Delta_j(t^*;\hat{\boldsymbol{\eta}}_j^{(k)})/\partial \boldsymbol{\eta}_j$ is the $p \times 1$ vector which is the first derivative of the function $\Delta_j(t^*;\boldsymbol{\eta}_j)$ with respect to the vector $\boldsymbol{\eta}_j$ evaluated at $\hat{\boldsymbol{\eta}}_j^{(k)}$. In the calculation of $\mathcal{I}_j^{(k)}$, a consistent estimate $\hat{\Sigma}_j^{(k)}$ can be substituted in place of the covariance matrix $\Sigma_j^{(k)}$. In practice, the MLE $\hat{\boldsymbol{\eta}}_j^{(k)}$ and an estimate of the covariance matrix can be calculated using the R package JM by Rizopoulos [8].

The value of the truncation time t^* is important for defining the estimand of the clinical trial and we believe that t^* should be chosen to be clinically meaningful. Contrastingly, Royston and Parmar [10] suggest taking t^* as the value that minimises the expected sample size given the recruitment time and minimum follow-up time. For our simulation study in the main text, each trial has recruitment roughly 2 years and final analysis time at roughly 5 years. To ensure that the method is robust to model misspecifications, it is important to avoid extrapolation of the RMST estimate beyond analysis time. Hence, we suggest using RMST at time $t^* = 5$ which is primarily chosen to be clinically meaningful and secondly meets the suggestions.

To summarise the overall effect of treatment on survival incorporating both treatment parameters b_{2j} and θ_j, we test the null hypothesis

$$H_{0j} : \Delta_j(5;\boldsymbol{\eta}_j) \le 0 \qquad \text{for } j = 1, 2, F.$$

To do so, we define RMST estimates $\Delta_j(5;\hat{\boldsymbol{\eta}}_j^{(k)})$, information levels $\mathcal{I}_j^{(k)}$ and Z-statistics for each $k = 1, \ldots, K$ and $j = 1, 2, F$.

A.3 Discussion of the use of RMST for enrichment trials

There are benefits to using the RMST methodology to incorporate a causal effect ef treatment acting through the biomarker. However, this method is accompanied by additional challenges.

The RMST methodology suffers from the complication relating to the choice of the truncation time t^*. The choice of t^* is not always well described and small differences can result in large changes in the operating characteristics of a trial. Therefore, t^* requires careful consideration and planning. The RMST methodology relies on finding maximum likelihood estimates and the model is overparameterised when any parameter is equal to zero. In such a case, the resulting covariance matrix is often not positive semi-definite and the analysis cannot be performed.

The advantage of the conditional score method is that no assumptions are required for either the distribution of the random effects or the functional form of the baseline hazard function. The RMST method does allow us to make inferences which leverage information about the treatment effect in the longitudinal data, however we have found that in comparison to the conditional score method, there is not much to be gained by doing so.

Table 1: Comparison of the p-value combinaton approach and the conditional score method.

		Median survival	$\mathbb{P}$(select S_1)		FWER		Power	
η	b_2	difference	Conditional score	P-value combination	Conditional score	P-value combination	Conditional score	P-value combination
$\mathbf{0}$	$\mathbf{0}$	0	0.175	0.181	0.024	0.014	-	-
$\mathbf{-0.5}$	$\mathbf{0}$	0.154	0.597	0.163	-	-	0.919	0.199
$\mathbf{-0.5}$	$\mathbf{-0.275}$	0.255	0.587	0.287	-	-	0.914	0.306
$\mathbf{-0.5}$	$\mathbf{-0.55}$	0.385	0.594	0.430	-	-	0.919	0.467
$\mathbf{0}$	$\mathbf{-0.55}$	0.154	0.173	0.446	-	-	0.083	0.168
$\mathbf{-0.25}$	$\mathbf{-0.55}$	0.265	0.386	0.444	-	-	0.489	0.293
$\mathbf{-0.5}$	$\mathbf{-0.55}$	0.385	0.594	0.430	-	-	0.919	0.467

A.4 P-value combination approach

When it is believed that treatment affects the longitudinal biomarker trajectory, another approach for designing an enrichment trial is to implement the p-value combination approach as described by Friede et al. [2]. This design makes interim subgroup selection decisions based on the longitudinal biomarker alone and hypothesis decisions at the final analysis using the survival outcome only. We compare the methods in the main paper which are based on a joint model with the p-value combination approach.

As discussed by Friede et al. [2], in order to implement the p-value combination approach, the “p-clud" conditional must be satisfied. This requires the distribution of the p-value from stage 1 and the conditional distribution of the p-value from stage 2 given stage 1 p-value to be stochastically no smaller than $U(0, 1)$. When time-to-event outcomes are considered, the p-clud condition is often violated due to introduction of bias from overlapping time periods. Jenkins et al. [3] address this problem by forming test statistics based on patients recruited to stage 1 and stage 2 separately. We make this adjustment when implementing the p-value combination approach in our simulations.

In what follows, we perform a simulation study under the worst case possible for the joint modeling method where the treatment acts mainly through the biomarker and there are small changes to the *additional* effect of treatment in the time-to-event outcome. For completeness, we also compare the two methods when treatment has a direct effect on survival with moderate magnitude. To make results comparable across cases, the values of η and b_2 in model (1) are chosen to give similar differences in median survival times between treatment and control groups. Table 1 shows the median survival differences for each parameter combination. For example, when treatment acts solely through the longitudinal data ($\eta = 0, b_2 = -0.55$), median survival times are 1.154 years and 1 years for patient receiving the experimental and control treatments respectively. These are the same median survival times as when there is no effect of treatment in the biomarker ($\eta = -0.5, b_2 = 0$). Further, it is known that the p-value combination method is most powerful when the short-term and long-term endpoints are well correlated. To achieve this we fixed parameter values $\gamma = 1.2, \sigma^2 = 0.25$ and $\phi = 7.5$ for simulating data as these parameters give correlation 0.65 between endpoints which was the largest from our grid of parameter values. For each simulation with $N = 10^4$ replicates, the interim analysis is performed when $d_1^{(1)} = 49$ events have been observed in subgroup S_1 and the final analysis is performed after a total of $d^{(2)} = 211$ events in the selected subgroup.

Table 1 compares the operating characteristics of the p-value combination approach with the conditional score method of Section 3.3 of the main text. Under H_0, the probability of selecting S_1 at the interim analysis is roughly the same between the two methods. In each case, the threshold selection rule with the same threshold value ζ is being applied to a $Z-$statistic which is $N(0, 1)$ distributed under H_0. Therefore, the selection probabilities are roughly equal. In line with the results of Friede et al. [2], the FWER is controlled at level $\alpha = 0.025$ using the p-value combination approach but is somewhat conservative.

Under H_A with $\eta = -0.5$, the trial has been designed to select subgroup S_1 at the interim analysis with probability 0.6 using the joint modeling approach and we see that this is as expected with smaller η magnitude leading to lower probability of selecting the subgroup which truly benefits from treatment. The probability of selecting S_1 increases with the magnitude of b_2 under the p-value combination approach but is smaller than the conditional score method for a similar increase in the difference in median survival time between the experimental treatment versus control. We see a similar pattern in power for the conditional score method; this is roughly 0.9 whenever $\eta = -0.5$ as per the design and reduces for smaller magnitude in η. Power of the p-value combination approach is affected by both parameters η and b_2 as the decision regarding H_0 is determined using the survival outcome. The worst case scenario for the joint modeling

Table 2: Number of events required $d_1^{(1)}$ at the interim analysis in subgroup S_1 for different threshold selection rules. Number of events calculated to satisfy FWER 0.025 and power 0.9.

$\mathbb{P}(W=1;\mathbf{\Theta}_A)/\mathbb{P}(W=F;\mathbf{\Theta}_A)$	0.1	0.2	0.3	0.4	0.5	0.6	0.7	0.8
0.1	1	1	1	1	1	1	1	1
0.2	1	1	1	1	1	1	6	-
0.3	4	2	2	3	6	15	-	-
0.4	15	10	10	15	27	-	-	-
0.5	30	24	28	41	-	-	-	-
0.6	51	47	59	-	-	-	-	-
0.7	80	85	-	-	-	-	-	-
0.8	126	-	-	-	-	-	-	-

Table 3: Total number of events $d^{(2)}$ in the selected subgroup at the final analysis for different threshold selection rules. Number of events calculated to satisfy FWER 0.025 and power 0.9.

$\mathbb{P}(W=1;\mathbf{\Theta}_A)/\mathbb{P}(W=F;\mathbf{\Theta}_A)$	0.1	0.2	0.3	0.4	0.5	0.6	0.7	0.8
0.1	142	189	200	204	207	208	209	211
0.2	174	191	199	204	206	209	213	-
0.3	168	188	197	203	209	217	-	-
0.4	165	187	199	208	220	-	-	-
0.5	170	192	207	224	-	-	-	-
0.6	184	206	229	-	-	-	-	-
0.7	207	235	-	-	-	-	-	-
0.8	248	-	-	-	-	-	-	-

method is when the treatment effect acts completely through the longitudinal data so that $\eta = 0$ and $b_2 = -0.55$ and basing subgroup selection and hypothesis testing decisions on an estimate of η will be futile. We see however, that this is the only case where power is higher for the p-value combination approach.

Overall, the joint modeling approach that we have proposed is more efficient than the p-value combination method with higher probability of selecting the subpopulation which is truly benefiting and higher power. We believe this is because of two reasons. The joint modeling method makes full use of all the information at each analysis whereas the p-value combination method neglects useful information at each stage; ignoring available survival outcomes at the interim and ignoring biomarker observations at the final analysis. However, the benefits of this method are likely to be more apparent in situations where very few, if any, survival outcomes are observed at the interim analysis. Friede et al.[2] describe this in the setting of a seamless Phase II/III trial whereas we have considered a Phase III enrichment trial where large numbers of patients are recruited and it is reasonable to assume that roughly 30 events will be observed at the interim analysis. Hence, to utilise the available information in the most efficient manner, the joint modeling approach is best suited for this setting.

B Details for the enrichment trial design

B.1 Sensitivity analyses for the threshold selection rule

The threshold selection rule is used to decide which subgroup, if any, to continue the trial in at the interim analysis. The rule depends on a parameter ζ, the value of which is chosen to satisfy some operating characteristics. Sensitivity analyses for different values of $\mathbb{P}(W=1;\mathbf{\Theta}_A)$ and $\mathbb{P}(W=F;\mathbf{\Theta}_A)$ are now presented. From the simulation result in Section 5 of the main document, it is known that the joint modeling approach using the conditional score estimator produces correct selection probabilities and controls Type 1 and Type 2 error rates. The outcomes of interest are therefore the number of events $d_1^{(1)}$ and $d^{(2)}$ required to satisfy some power requirements.

Table 2 shows the number of required events $d_1^{(1)}$ in subgroup S_1 at the interim analysis. The choice of $\mathbb{P}(W=1;\mathbf{\Theta}_A)$ is influential with high probability of selecting S_1 requiring large number of events in this subgroup as expected. In order for the asymptotic assumptions of the conditional score estimator to hold, roughly 30 events in each subgroup are required. Therefore, it is recommended that the trial is designed with $\mathbb{P}(W=1;\mathbf{\Theta}_A) \geq 0.5$. Further, $d_1^{(1)}$ appears to increase by a small margin as the probability of continuing the trial in the full population increases.

Table 3 shows the total number of events $d^{(2)}$ in the selected group at the final analysis. This increases with both $\mathbb{P}(W = 1; \boldsymbol{\Theta}_A)$ and $\mathbb{P}(W = F; \boldsymbol{\Theta}_A)$ as expected.

B.2 Joint density function in the full population

To compute the joint density $f_{Z_W^{(1)},W}(z_F^{(1)}, F; \boldsymbol{\Theta})$ we shall decompose $Z_F^{(1)}$ into the sum of two independent normal random variables and apply the convolution formula for probability density functions. Let $Z_F^{(1)} = X_1 + X_2$ where X_1 and X_2 are normally distributed random variables given by

$$X_1 = \lambda\sqrt{\mathcal{I}_F^{(1)}}\hat{\theta}_1^{(1)} \sim N\left(\lambda\sqrt{\mathcal{I}_F^{(1)}}\mu_1, \lambda^2\mathcal{I}_F^{(1)}/\mathcal{I}_1^{(1)}\right)$$
$$X_2 = \lambda\sqrt{\mathcal{I}_F^{(1)}}\hat{\theta}_2^{(1)} \sim N\left(\lambda\sqrt{\mathcal{I}_F^{(1)}}\mu_2, \lambda^2\mathcal{I}_F^{(1)}/\mathcal{I}_2^{(1)}\right).$$

The threshold selection criterion is transformed so that the constraint $Z_1 > \zeta$ implies that $X_1 > \lambda\sqrt{\mathcal{I}_F^{(1)}}\zeta/\sqrt{\mathcal{I}_1^{(1)}}$ and the case $Z_2 > \zeta$ is transformed to $X_2 > \lambda\sqrt{\mathcal{I}_F^{(1)}}\zeta/\sqrt{\mathcal{I}_2^{(1)}}$. By the convolution formula for probability density functions, we have

$$f_{Z_W^{(1)}|W}(z_F^{(1)}|W = F; \boldsymbol{\Theta}) = \int_{-\infty}^{\infty} f_{X_1|W}(u|W = 1; \boldsymbol{\Theta}) f_{X_2|W}(x_2 - u|W = 1; \boldsymbol{\Theta}) du$$
$$= \int_{-\infty}^{\infty} \frac{\sqrt{\mathcal{I}_1^{(1)}\mathcal{I}_2^{(1)}}\phi\left(\frac{\sqrt{\mathcal{I}_1^{(1)}}(u-\lambda\sqrt{\mathcal{I}_F^{(1)}})}{\lambda\sqrt{\mathcal{I}_F^{(1)}}}\right)\phi\left(\frac{\sqrt{\mathcal{I}_2^{(1)}}(z_F^{(1)}-u-(1-\lambda)\sqrt{\mathcal{I}_F^{(1)}})}{(1-\lambda)\sqrt{\mathcal{I}_F^{(1)}}}\right)}{\lambda(1-\lambda)\mathcal{I}_F^{(1)}\left[1-\Phi\left(\zeta-\mu_1\sqrt{\mathcal{I}_1^{(1)}}\right)\right]\left[1-\Phi\left(\zeta-\mu_2\sqrt{\mathcal{I}_2^{(1)}}\right)\right]} du$$

In the main paper, we defined $f_{Z_W^{(1)},W}\left(z_w^{(1)}, w; \boldsymbol{\Theta}\right) = \mathbb{P}\left(W = w; \boldsymbol{\Theta}\right) f_{Z_W^{(1)}|W}\left(z_w^{(1)}|W = w; \boldsymbol{\Theta}\right)$. Therefore, combining this with the threshold selection criteria, we have

$$f_{Z_W^{(1)},W}(z_F^{(1)}, F; \boldsymbol{\Theta}) = \frac{\sqrt{\mathcal{I}_1^{(1)}\mathcal{I}_2^{(1)}}}{\lambda(1-\lambda)\mathcal{I}_F^{(1)}} \int_{-\infty}^{\infty} \phi\left(\frac{\sqrt{\mathcal{I}_1^{(1)}}(u-\lambda\sqrt{\mathcal{I}_F^{(1)}})}{\lambda\sqrt{\mathcal{I}_F^{(1)}}}\right)\phi\left(\frac{\sqrt{\mathcal{I}_2^{(1)}}(z_F^{(1)}-u-(1-\lambda)\sqrt{\mathcal{I}_F^{(1)}})}{(1-\lambda)\sqrt{\mathcal{I}_F^{(1)}}}\right) du.$$

B.3 Proof of strong control of the FWER

We now show that the threshold selection rule combined with an error spending test controls the FWER in the strong sense. We shall use the following definitions

$$S_1(\theta_1, \theta_2) = \left\{Z_1^{(1)} > \zeta - \theta_1\sqrt{\mathcal{I}_1^{(1)}} \cap Z_2^{(1)} \leq \zeta - \theta_2\sqrt{\mathcal{I}_2^{(1)}}\right\}$$
$$S_2(\theta_1, \theta_2) = \left\{Z_1^{(1)} \leq \zeta - \theta_1\sqrt{\mathcal{I}_1^{(1)}} \cap Z_2^{(1)} > \zeta - \theta_2\sqrt{\mathcal{I}_2^{(1)}}\right\}$$
$$S_F(\theta_1, \theta_2) = \left\{Z_1^{(1)} > \zeta - \theta_1\sqrt{\mathcal{I}_1^{(1)}} \cap Z_2^{(1)} > \zeta - \theta_2\sqrt{\mathcal{I}_2^{(1)}}\right\}$$
$$A_j^{(k)}(\theta_j) = \left\{a_k - \theta_j\sqrt{\mathcal{I}_j^{(k)}} < Z_j^{(k)} < b_k - \theta_j\sqrt{\mathcal{I}_j^{(k)}}\right\}$$
$$B_j^{(k)}(\theta_j) = \left\{Z_j^{(k)} > b_k - \theta_j\sqrt{\mathcal{I}_j^{(k)}}\right\}.$$

The sets $S_1(\theta_1, \theta_2), S_2(\theta_1, \theta_2), S_F(\theta_1, \theta_2)$ describe the selection criteria and the sets $A_j^{(k)}(\theta_j)$ and $B_j^{(k)}(\theta_j)$ represent the accept and reject regions of the hypothesis test H_j for $j = 1, 2, F$.

Suppose that $\mathcal{L} = \{j = 1, 2, F|\theta_j \leq 0\}$ is the set of indices corresponding to *true* null hypotheses $H_{0,j}, (j = 1, 2, F)$. Let $R_j(\theta_j)$ be the event that H_{0j} is rejected and let $\bar{R}(\theta_1, \theta_2)$ be the event that at least one true $H_{0,j}$ is rejected. These

are given by

$$R_j(\theta_j) = \bigcup_{k=1}^{K} \left[\left\{ \bigcap_{m=1}^{k-1} A_j^{(m)}(\theta_j) \right\} \cap B_j^{(k)}(\theta_j) \right]$$
$$\bar{R}(\theta_1, \theta_2) = \bigcup_{j \in \mathcal{L}} \left(S_j(\theta_1, \theta_2) \cap R_j(\theta_j) \right).$$

In what follows, we aim to show that $\bar{R}(\theta_1, \theta_2) \subseteq \bar{R}(0, 0)$, which leads to showing that FWER is maximized under the global null. To do so, we impose the following condition.

Conditions 1. *The treatment effect in the full population, $\theta_F = \lambda\theta_1 + (1-\lambda)\theta_2$, is non-negative.*

We note here the similarity between Condition 1 and the condition in the proof by Magnusson and Turnbull[7], where the authors make the assumption that treatment effects θ_j cannot be negative for any j. Magnusson and Turnbull[7] argue that treatment effects of opposite sign are "rare and highly implausible". Our condition is not as restrictive, since treatment effects other than θ_F are allowed to be negative. This condition ensures that the subgroup selected does not differ under scenarios (θ_1, θ_2) and $(0, 0)$. We believe that, with moderate information levels, these unaccounted events are so unlikely that they will not affect the FWER. Without this assumption however, it is possible to show that asymptotically the FWER is protected in the strong sense. As n increases, the information levels increase and it can be seen that $\limsup_{n\to\infty} S_i(\theta_1, \theta_2) \cap S_j(0, 0) = \emptyset$ for any $i \neq j$. Under Condition 1, there are four possibilities for the configuration of $\boldsymbol{\Theta} = (\theta_1, \theta_2)$ and these are

1. $\theta_1 = \theta_2 = \theta_F = 0$
2. $\theta_1 \leq 0, \theta_2 > 0, \theta_F > 0$
3. $\theta_1 > 0, \theta_2 \leq 0, \theta_F > 0$
4. $\theta_1 > 0, \theta_2 > 0, \theta_F > 0$

We are now equipped to prove that the threshold selection rule combined with an error spending test controls the FWER in the strong sense.

Theorem 1. *For global null hypothesis H_G and any $\boldsymbol{\Theta} = (\theta_1, \theta_2)$ such that Condition 1 holds, we have*

$$\mathbb{P}(\textit{Reject at least one true } H_j | \boldsymbol{\Theta}) \leq \mathbb{P}(\textit{reject at least one } H_j | H_G).$$

Proof. For this proof, we first show that $\bar{R}(\theta_1, \theta_2) \subseteq \bar{R}(0, 0)$ for each of the four cases which were possible under Condition 1. For the first case, we have that $\theta_1 = \theta_2 = \theta_F = 0$ which is equivalent to the global null and we have $\bar{R}(\theta_1, \theta_2) = \bar{R}(0, 0)$.

For case 2, when $\theta_1 \leq 0, \theta_2 > 0, \theta_F > 0$, the event that at least one true $H_{0,j}$ is rejected is $\bar{R}(\theta_1, \theta_2) = S_1(\theta_1, \theta_2) \cap R_1(\theta_1)$ and hence we show that $S_1(\theta_1, \theta_2) \cap R_1(\theta_1) \subseteq S_1(0, 0) \cap R_1(0)$. Suppose that $x = (Z_1^{(1)}, \ldots, Z_1^{(K)}, Z_2^{(1)}, \ldots, Z_2^{(K)}) \in S_1(\theta_1, \theta_2) \cap R_1(\theta_1)$, so that $Z_1^{(1)} > \zeta - \theta_1\sqrt{\mathcal{I}_1^{(1)}}$ and $Z_2^{(1)} \leq \zeta - \theta_2\sqrt{\mathcal{I}_2^{(1)}}$. But $\theta_1 \leq 0$ and $\theta_2 > 0$ so $Z_1^{(1)} > \zeta$ and $Z_2^{(1)} \leq \zeta$ so $x \in S_1(0, 0)$. Following the work of Magirr et al.[6], we also have that $x \in \bigcup_{k=1}^{K} \left[\left\{ \bigcap_{m=1}^{k-1} A_1^{(m)}(\theta_1) \right\} \cap B_1^{(k)}(\theta_1) \right]$. For some $k \in \{1, \ldots, K\}$, $Z_1^{(k)} \in B_1^{(k)}(\theta_1)$ and $Z_1^{(m)} \in A_1^{(m)}(\theta_1)$ for $m = 1, \ldots, k-1$. $Z_1^{(k)} \in B_1^{(k)}(\theta_1)$ implies that $Z_1^{(k)} \in B_1^{(k)}(0)$ and $Z_1^{(m)} \in A_1^{(m)}(\theta_1)$ implies that $Z_1^{(m)} \in A_1^{(m)}(0) \cup B_1^{(m)}(0)$ for $m = 1, \ldots, k-1$. Therefore $x \in \bigcup_{k=1}^{K} \left[\left\{ \bigcap_{m=1}^{k-1} A_1^{(m)}(0) \right\} \cap B_1^{(k)}(0) \right]$. Hence, we have the result $\bar{R}(\theta_1, \theta_2) = \bar{R}(0, 0)$.

Case 3 can be shown by exactly the same argument as for case 2, replacing all indices $j = 1$ with $j = 2$. Finally, Case 4 is trivial since this is the case where none of the hypotheses $H_{0j}, (j = 1, 2, F)$ are true. Hence the event that at least one true $H_{0,j}$ is rejected is $\bar{R}(\theta_1, \theta_2) = \emptyset$. In each of the four cases we have $\bar{R}(\theta_1, \theta_2) \subseteq \bar{R}(0, 0)$ and therefore

$$\begin{aligned} \mathbb{P}\{\text{Reject at least one true } H_j | \theta_1, \theta_2\} &= \mathbb{P}\{\bar{R}(\theta_1, \theta_2)\} \\ &\leq \mathbb{P}\{\bar{R}(0, 0)\} \\ &= \mathbb{P}\{\text{Reject at least one true } H_j | H_0\}. \end{aligned}$$

□

C Additional results from simulation studies

C.1 Robustness of the conditional score estimator

The conditional score estimator provides a method for estimating the treatment effect in the joint model. In the main paper, we have made some distributional assumptions which we now check. In particular, we consider the asymptotic properties of the estimator under small sample sizes and the robustness of the assumption that error terms are independent.

In Table 1 of the main paper, we have presented the number of events required in the biomarker positive subgroup at the interim analysis and have found that using these numbers results in Type 1 error rates being controlled at level $\alpha = 0.025$ and power being close to $1 - \beta = 0.9$ as required. To achieve such control, the asymptotic assumptions must therefore hold for these sample sizes. Figure 1 shows histograms and QQ-plots for the treatment effects in a simulation study with 10^4 replicates. Each simulation recruited 100 patients and the interim analysis was performed when 20 events in total across treatment groups were observed in subgroup S_1. We considered the distribution of $Z_1^{(1)}$ under H_0 and H_A since this is assumed to have unit variance in each case. Under H_0, $Z_1^{(1)}$ has mean slightly below zero, but this is in keeping with the results of Langner et al.[5]. Otherwise, $Z_1^{(1)}$ follows a $N(0, 1)$ distribution as expected within acceptable range. Under H_A we see a similar pattern that the assumed distribution is appropriate, with discrepancies at an acceptable level. We also assessed the conditional score method for 10 events in each subgroup but found this to give a poor fit to the normal distribution. Therefore, we conclude that a minimum of 20 events in each subgroup is enough to rely on asymptotic assumptions and ensure that Type 1 and Type 2 error rates are controlled.

Asymptotic properties of the conditional score estimator are reliant on the assumption of independent error terms. Tsiatis and Davidian[11] state that “normality and independence may be a reasonable assumption for within-subject error in continuous covariates" and we now investigate the robustness of the conditional score estimator when the error terms are truly correlated. To do so, residuals were simulated according to an AR(1) process. Figure 2 show a comparison between the estimates calculated using the conditional score method when error terms are simulated independently versus when they are simulated according to an AR(1) process. For this comparison, the auto-correlation parameter is set as $\rho = 0.95$ to emulate the case where error terms are correlated to an extreme degree. $Z_1^{(1)}$ appears to be approximately normally distributed when error terms are simulated from an AR(1) process highlighting that the conditional score estimator is robust to the assumption of independent error terms.

C.2 Alternative outcome measures

We present the results from the simulation study in the results section of the main paper. To recap, for one simulation; generate a dataset of patients from the joint model, then subgroup selection and decisions about H_0 are performed after $d_1^{(1)}$ and $d^{(2)}$ events have been observed. During each simulation run, all four methods are evaluated using the same dataset and after the same number of events. This is so that differences in the trial results can be attributed to the analysis methodology and not trial design features. The simulations are repeated $N = 10^4$ times for each set of parameter values.

FWER for the simulation studies are shown in Web Table 4. Results confirm that the FWER are close to 0.025 for $N = 10^4$ simulations.

Selection probabilities, denoted $\mathbb{P}(\text{Select } S_1)$ in Web Table 5, are calculated as the proportion of simulations which select subgroup S_1. This is an appropriate summary metric since S_1 is the subgroup which truly benefits from the experimental treatment. The value of $d_1^{(1)}$ has been calculated to ensure $\mathbb{P}(\text{Select } S_1) = 0.6$ using the conditional score method. Web Table 5 confirms that the probabilities are close to 0.6 for $N = 10^4$ simulations. The value of $d^{(2)}$, has been calculated with reasonable accuracy since power is suitably close to 0.9 for $N = 10^4$ in all cases for the conditional score method. The alternative methods do not attain the desired selection probabilities because the trial is designed using the conditional score analysis method. Generally, the Cox method has lower selection probabilities than the conditional score method and the Cox with biomarker has higher selection probabilities than the Conditional score method. These results become more extreme for increases in γ.

We also present the expected number of hospital visits per patient and the expected stopping time for each method. In Web Table 6, the expected number of hospital visits is calculated as the mean number of longitudinal observations across all patients enrolled in the study across all simulations and the expected stopping time is the average study duration in years. It is challenging to make comparisons between methods for these outcomes. This is because the number of patients is not the same between methods due to capability of each method to select the correct subgroup. For example, it would appear at first sight that the simple Cox model method is most efficient because it results in the

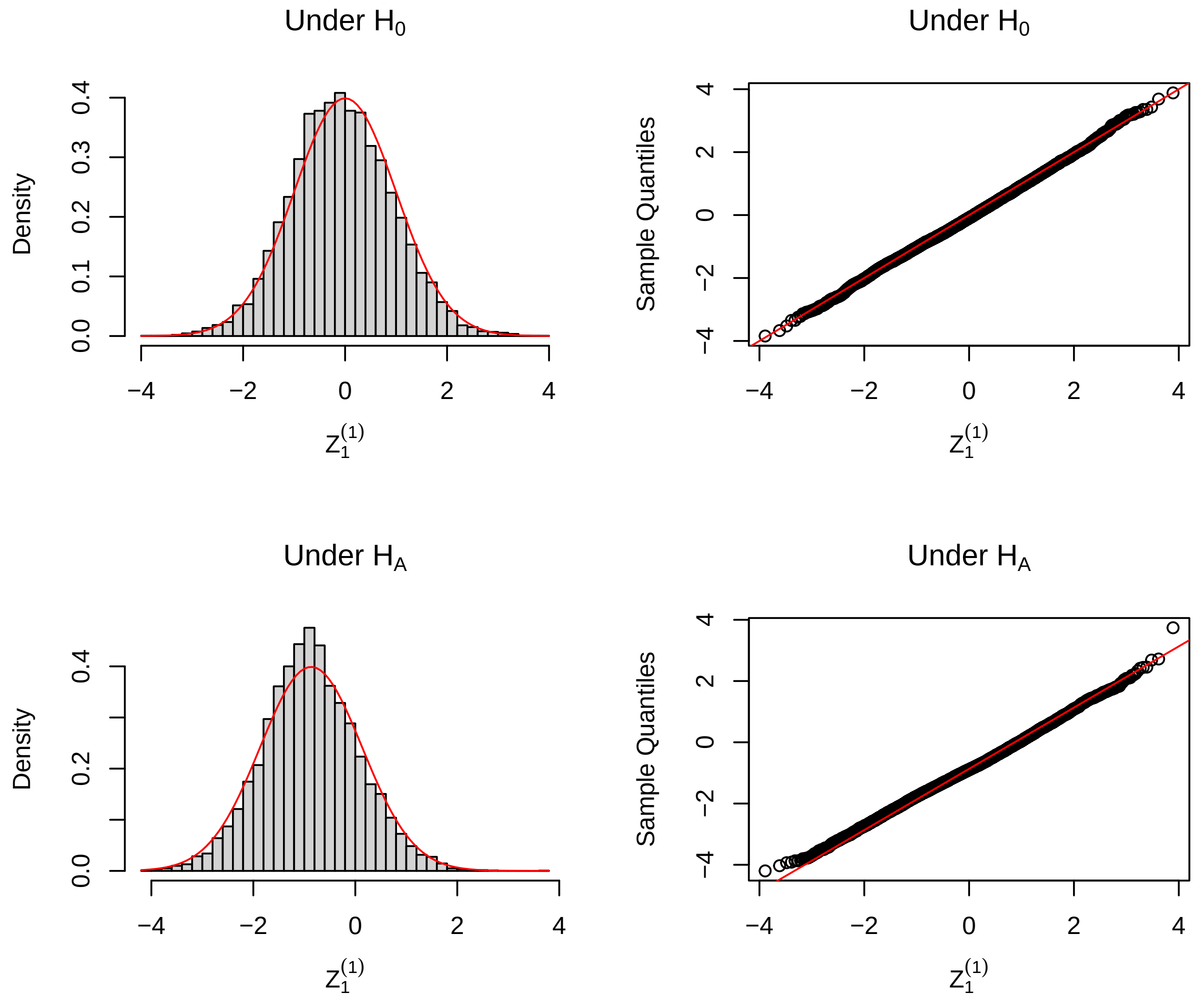

Figure 1: Histograms and QQ-plots for the treatment effect estimate using the conditional score estimator in a simulation study with $N = 10^4$ replicates. Each estimate is calculated with 20 events out of 100 recruited patients. Red lines represent the normal distribution with unit variance which the estimates are assumed to follow.

Table 4: Family wise error rates (FWER) for each method.

			FWER		
γ	σ^2	ϕ_2	Conditional score	Cox	Cox with biomarker
**0**	**0.25**	**5**	0.026	0.024	0.025
**0.4**	**0.25**	**5**	0.025	0.026	0.026
**0.8**	**0.25**	**5**	0.024	0.024	0.019
**1.2**	**0.25**	**5**	0.026	0.018	0.014
**0.8**	**0**	**5**	0.028	0.025	0.024
**0.8**	**0.25**	**5**	0.024	0.024	0.019
**0.8**	**1**	**5**	0.020	0.023	0.011
**0.8**	**2.25**	**5**	0.025	0.029	0.014
**0.8**	**0.25**	**0**	0.026	0.025	0.018
**0.8**	**0.25**	**2.5**	0.021	0.022	0.016
**0.8**	**0.25**	**5**	0.024	0.024	0.019
**0.8**	**0.25**	**7.5**	0.026	0.025	0.019

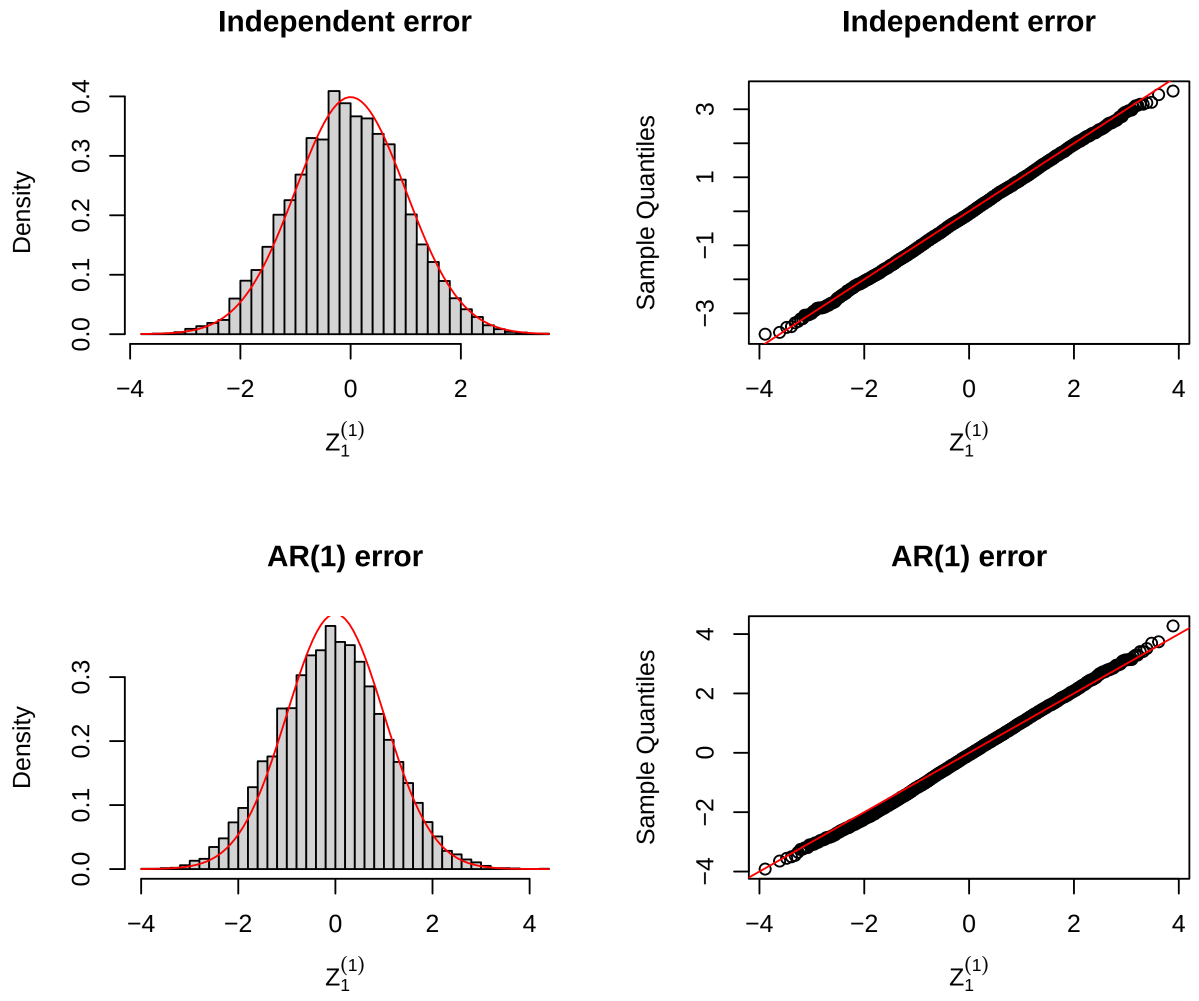

Figure 2: Comparison of independent error terms compared to error simulated using an AR(1) process. Histograms and QQ-plots for a simulation study using $N = 10^4$ replicates. Each estimate is calculated with 100 events out of 200 recruited patients. Red lines represent the normal distribution with unit variance which the estimates are assumed to follow.

shortest average stopping time, for all cases. However, we find that this method has a relatively low chance of selecting any subgroup and often the trial stops at the first interim analysis to declare that the treatment is inefficacious in all subgroups. We have not included the number of hospital visits per patient for the simple Cox model and this is to highlight an advantage of the method that we do not need to collect the longitudinal data at all in this case. It is clear, however, that the trends in number of hospital visits and stopping times all follow the same structure as the trends in the probability of selecting subgroup S_1. Hence, for large γ, ϕ_2 and σ, we require patients to have more regular blood tests and also the trial will take longer to reach a decision.

D Details of the estimation of treatment effect estimates in alternative Cox proportional hazards models

D.1 Cox proportional hazards model

We now give an overview of how treatment effect estimates and information levels are calculated for each of the alternative Cox proportional hazards models. As a reminder, we introduce the model again. Denote $h_{0j}(t)$ as the

Table 5: Selection probabilities and power for each method.

γ	σ^2	ϕ_2	$\mathbb{P}$(select S_1) Conditional score	Cox	Cox with biomarker	Power Conditional score	Cox	Cox with biomarker
**0**	**0.25**	**5**	0.588	0.610	0.604	0.908	0.915	0.914
**0.4**	**0.25**	**5**	0.636	0.541	0.626	0.909	0.616	0.897
**0.8**	**0.25**	**5**	0.625	0.431	0.621	0.907	0.570	0.887
**1.2**	**0.25**	**5**	0.611	0.363	0.618	0.903	0.440	0.862
**0.8**	**0**	**5**	0.628	0.424	0.619	0.904	0.560	0.897
**0.8**	**0.25**	**5**	0.625	0.431	0.621	0.907	0.570	0.887
**0.8**	**1**	**5**	0.637	0.438	0.627	0.905	0.590	0.851
**0.8**	**2.25**	**5**	0.631	0.458	0.631	0.906	0.610	0.787
**0.8**	**0.25**	**0**	0.628	0.503	0.617	0.910	0.780	0.915
**0.8**	**0.25**	**2.5**	0.612	0.433	0.600	0.907	0.620	0.883
**0.8**	**0.25**	**5**	0.625	0.431	0.621	0.907	0.570	0.887
**0.8**	**0.25**	**7.5**	0.643	0.420	0.627	0.909	0.480	0.858

Table 6: Expected number of hospital visits per patien and expected stopping times for each method.

γ	σ^2	ϕ_2	$\mathbb{E}$(number of hospital visits per patient) Conditional score	Cox	Cox with biomarker	$\mathbb{E}$(stopping time) Conditional score	Cox	Cox with biomarker
**0**	**0.25**	**5**	11.1	-	11.3	2.96	2.98	2.98
**0.4**	**0.25**	**5**	12.0	-	11.9	3.32	3.22	3.31
**0.8**	**0.25**	**5**	11.9	-	11.9	3.41	3.11	3.42
**1.2**	**0.25**	**5**	11.7	-	11.7	3.57	3.13	3.57
**0.8**	**0**	**5**	11.8	-	11.8	3.26	3.00	3.30
**0.8**	**0.25**	**5**	11.9	-	11.9	3.41	3.11	3.42
**0.8**	**1**	**5**	12.4	-	12.0	3.96	3.52	3.87
**0.8**	**2.25**	**5**	12.5	-	12.1	4.56	3.86	4.27
**0.8**	**0.25**	**0**	11.3	-	11.2	3.07	2.93	3.05
**0.8**	**0.25**	**2.5**	11.6	-	11.5	3.23	2.98	3.23
**0.8**	**0.25**	**5**	11.9	-	11.9	3.41	3.11	3.42
**0.8**	**0.25**	**7.5**	12.1	-	11.9	3.47	3.11	3.46

baseline hazard function, θ_j the treatment effect and ψ_{ji} as the treatment indicator that patient i in subgroup $j = 1, 2$ receives the new treatment. Then the hazard function for the survival model is given by

$$h_{ji}(t) = h_{0j}(t) \exp\{\theta_j \psi_{ji}\}. \tag{4}$$

Let $t_{ji}^{(k)}$ be the observed event time and let $\delta_{ji}^{(k)}$ be the observed censoring indicator for patient i in subgroup $j = 1, 2$ at analysis k. Then $Y_{ji}^{(k)}(t) = \mathbb{I}\{t_{ji}^{(k)} \geq t\}$ is the at-risk process and $dN_{ji}^{(k)}(t) = \mathbb{I}\{t \leq t_{ji}^{(k)} < t + dt, \delta_{ji}^{(k)} = 1\}$ is the counting process. As in Jennison and Turnbull[4], the function $E_j^{(k)}(u, \theta_j)$ and the score function $U_j^{(k)}(\theta_j)$ at analysis k are given by

$$\begin{aligned} E_j^{(k)}(t, \theta_j) &= \frac{\sum_{i=1}^{n_j} \psi_{ji} \exp\{\theta_j \psi_{ji}\} Y_{ji}^{(k)}(t)}{\sum_{i=1}^{n_j} \exp\{\theta_j \psi_{ji}\} Y_{ji}^{(k)}(t)} \\ U_j^{(k)}(\theta_j) &= \int_0^{\tau_k} \sum_{i=1}^{n_j} \left(\psi_{ji} - E_j^{(k)}(t, \theta_j) \right) d\tilde{N}_{ji}^{(k)}(t). \end{aligned} \tag{5}$$

The function $U_j^{(k)}(\cdot)$ is a score function and θ_j can be estimated by solving the equation $U_j^{(k)}(\theta_j) = 0$ for θ_j. Let this estimate, at analysis k, be denoted by $\hat{\theta}_j^{(k)}$. By standard results by Jennison and Turnbull[4], the estimates $\hat{\theta}_j^{(k)}$ follow the CJD where the information level at analysis k is given by $\mathcal{I}_j^{(k)} = n_j \left[\partial U_j^{(k)}(\hat{\theta}_j^{(k)})/\partial\theta_j\right]^{-1}$.

D.2 Cox proportional hazards model with longitudinal data as a time-varying covariate

As a reminder, $W_{ji}(v_{ji1}), \ldots, W_{ji}(v_{jim_{ji}})$ are the observed values of the biomarker for patient i in subgroup j at times $v_{j-1}, \ldots, v_{jim_{ji}}$ and the definitions of the the at-risk process $Y_{ji}^{(k)}(t)$ and counting process function $dN_{ji}^{(k)}(u)$ are as in Section D.1. Let γ_j and θ_j be longitudinal data and treatment coefficients respectively, then the hazard function is given by

$$h_{ji}(t) = h_{j0}(t)\exp\{\gamma_j W_{ji}(t) + \theta_j \psi_{ji}\}. \tag{6}$$

The function $E_j^{(k)}(u, \cdot)$ and score statistic $U_j^{(k)}(\cdot)$ for this model are given by

$$E_j^{(k)}(t, \theta_j) = \frac{\sum_{i=1}^{n_j} \{W_{ji}(t), \psi_{ji}\}^T \exp\{\gamma_j W_{ji}(t) + \theta_j\psi_{ji}\} Y_{ji}^{(k)}(t)}{\sum_{i=1}^{n_j} \exp\{\gamma_j W_{ji}(t) + \theta_j\psi_{ji}\} Y_{ji}^{(k)}(t)}$$

$$U_j^{(k)}(\theta_j) = \int_0^{\tau_k} \sum_{i=1}^{n_j} \left(\{W_{ji}(t), \psi_{ji}\}^T - E_j^{(k)}(t, \theta_j)\right) dN_{ji}^{(k)}(t). \tag{7}$$

Both objects $E_j^{(k)}(u, \theta_j)$ and $U_j^{(k)}(\theta_j)$ are 2×1 dimensional vectors. To evaluate these objects, we will need to know $W_{ji}(t_{js})$ which is the value of the time-varying covariate for patient i in subgroup j, evaluated at the event time of patient s in subgroup j. For this model, $W_{ji}(\cdot)$ is a function of time and is known. For calculation purposes, for $t > v$, we shall set $W_{ji}(t)$ as $W_{ji}(v)$ where $v = max(v_{jim} | v_{jim} \leq t)$.

Again, the function $U_j^{(k)}(\cdot)$ is a score function and θ_j can be estimated by solving the equation $U_j^{(k)}(\theta_j) = 0$ for θ_j and the information level at analysis k can be calculated as $\mathcal{I}_j^{(k)} = n_j \left[\partial U_j^{(k)}(\hat{\theta}_j^{(k)})/\partial\theta_j\right]^{-1}$.

References

[1] Joseph L Doob. The limiting distributions of certain statistics. *The Annals of Mathematical Statistics*, 6(3): 160–169, 1935.

[2] T Friede, N Parsons, N Stallard, Susan Todd, E Valdes Marquez, J Chataway, and R Nicholas. Designing a seamless phase ii/iii clinical trial using early outcomes for treatment selection: an application in multiple sclerosis. *Statistics in medicine*, 30(13):1528–1540, 2011.

[3] Martin Jenkins, Andrew Stone, and Christopher Jennison. An adaptive seamless phase ii/iii design for oncology trials with subpopulation selection using correlated survival endpoints. *Pharmaceutical statistics*, 10(4):347–356, 2011.

[4] Christopher Jennison and Bruce W Turnbull. Group-sequential analysis incorporating covariate information. *Journal of the American Statistical Association*, 92(440):1330–1341, 1997.

[5] Ingo Langner, Ralf Bender, Rebecca Lenz-Tönjes, Helmut Küchenhoff, and Maria Blettner. Bias of maximum-likelihood estimates in logistic and cox regression models: a comparative simulation study. Technical report, Discussion Paper, 2003.

[6] Dominic Magirr, Thomas Jaki, and John Whitehead. A generalized dunnett test for multi-arm multi-stage clinical studies with treatment selection. *Biometrika*, 99(2):494–501, 2012.

[7] Baldur P Magnusson and Bruce W Turnbull. Group sequential enrichment design incorporating subgroup selection. *Statistics in medicine*, 32(16):2695–2714, 2013.

[8] Dimitris Rizopoulos. *Joint Models for Longitudinal and Time-to-event Data: With Applications in R*. London: Chapman and Hall/CRC, 2012.

[9] Patrick Royston and Mahesh KB Parmar. The use of restricted mean survival time to estimate the treatment effect in randomized clinical trials when the proportional hazards assumption is in doubt. *Statistics in medicine*, 30(19): 2409–2421, 2011.

[10] Patrick Royston and Mahesh KB Parmar. Restricted mean survival time: an alternative to the hazard ratio for the design and analysis of randomized trials with a time-to-event outcome. *BMC Medical Research Methodology*, 13 (1):152, 2013.

[11] Anastasios A Tsiatis and Marie Davidian. A semiparametric estimator for the proportional hazards model with longitudinal covariates measured with error. *Biometrika*, 88(2):447–458, 2001.
